# Can we prevent anxiety in adults with congenital heart disease with good parenting practices in childhood?

**DOI:** 10.1192/j.eurpsy.2023.1429

**Published:** 2023-07-19

**Authors:** C. Houchi

**Affiliations:** Psychiatrie et Addictologie, Université de Montréal, Montréal, Canada

## Abstract

**Introduction:**

Medical-technical advances are contributing to the increased life expectancy of children with congenital heart disease (CHD) (Ladouceur et al., 2021). As they grow up into adulthood, they face many challenges (eg. surgeries, hospitalizations, separations from family, cardiac symptoms, anxiety symptoms). It is known that some parenting practices like parental overprotection during childhood are associated with anxiety in the general population, but little is known in this population.

**Objectives:**

We aim to measure the contribution of parental practices (global; positive: warm care, consistent structure, autonomy support; and negative: overprotection) to explain variance in anxiety symptoms in adults with CHD, beyond sociodemographic and antecedents of pediatric hospitalisations.

**Methods:**

An observational cross-sectional study was conducted on 223 adults with CHD followed at the Montreal Heart Institute. We evaluated anxiety symptoms and retrospective parental practices using validated self-reported questionnaires, namely the *Hospital Anxiety and Depression Scale*, the *Parental Bonding Inventory*, the *Perceived Parental Autonomy Support* and the *Multidimensional Parental Structure Scale.* Sociodemographic and antecedents of pediatric hospitalisations information was collected from medical records and pediatric archives. Hierarchical multiple linear regression analyses were conducted.

**Results:**

The average age of our participants is 46 years and the majority (59 %) were female at birth. The median number of hospitalisation before 18 years old was two. 15 % presented severe anxiety symptoms (HADS-A ≥ 11), 17 % had moderate symptoms (HADS-A = 8-10), and 68 % had mild or no symptoms (HADS-A ≤ 7).

The inclusion of parenting practices significantly increased the proportion of variance explaining anxiety symptoms. They explained more variance (13%) than sociodemographic and pediatric hospitalisations combined (10%).

In this model, only positive parenting practices were significantly associated with anxiety, in contrast to parental overprotection.

When the parental practices were analyzed separately, positive practices (autonomy, care, and structure) were negatively associated with anxiety symptoms, while overprotection was positively associated with anxiety symptoms.

**Conclusions:**

Our results suggest that although our participants’ physical health may be limited by their CHD, the majority report a low anxiety scores. Further, parenting practices appear to be malleable predictors of anxiety. Beyond avoiding overprotective parenting style, positive and supportive parenting practices are potential targets for future initiatives to prevent anxiety symptoms in adults with CHD.

**Disclosure of Interest:**

None Declared

